# “Just a number” – larger-bodied women’s experiences of weight-centric and weight-inclusive maternity care: a national online photovoice study

**DOI:** 10.1186/s12884-026-09715-9

**Published:** 2026-08-03

**Authors:** Bec Jenkinson, Lauren Kearney, Ahlia Griffiths, Brianne Mackinnon, Sara B. Gill, Briony Hill, Renee Tome, Tina Siale, Kayla Barrow, Laura Hewett, Tanya Cawthorne, Jennifer Hocking, Jacqueline A Boyle, Leonie Callaway

**Affiliations:** 1 UQ Clinical Trials Capability (ULTRA) Team, School of Health and Rehabilitation Sciences, The University of Queensland, Brisbane, Australia; 2 School of Nursing, Midwifery and Social Work, The University of Queensland, Brisbane, Australia; 3 Royal Brisbane and Women’s Hospital, Brisbane, Australia; 4 Lived Experience Expert, Melbourne, Australia; 5 Health and Social Care Unit, School of Public Health and Preventive Medicine, Monash University, Melbourne, Australia; 6 Lived Experience Expert, Scenic Rim, Australia; 7 Lived Experience Expert, Representing Birth Trauma Australia, Melbourne, Australia; 8 Lived Experience Expert, Representing Lamaze Australia, Melbourne, Australia; 9 Australian Breastfeeding Association, Melbourne, Australia; 10 Health Systems and Equity Directorate, Eastern Health Clinical School, Monash University, Melbourne, Australia; 11 Royal Brisbane Clinical Unit, Faculty of Health, Medicine and Behavioural Sciences, The University of Queensland, Brisbane, Australia

**Keywords:** Weight Stigma, Weight prejudice, Preconception, Antenatal care, Birth, Postnatal care, Obesity, maternal, Photovoice, Participatory research, Consumer involvement

## Abstract

**Background:**

Weight stigma is a pervasive issue in healthcare, including maternity care, characterised by bias, discrimination, and exclusion. Experiencing weight stigma can negatively impact health outcomes, self-esteem, and engagement with care. While existing literature documents the nature and consequences of weight stigma in maternity care, few studies have explored solutions or involved women with lived experience. This study aimed to investigate larger-bodied women’s experiences of weight-centric and weight-inclusive maternity care across the preconception, antenatal, intrapartum, and postnatal periods, as part of a broader program to co-create Best Practice Principles for Weight-Inclusive Maternity Care.

**Methods:**

A consumer-led, national online photovoice study was co-designed and co-facilitated by a multidisciplinary team of lived experience experts and academic researchers. Qualitative data—including transcripts, images, and discussions—were analysed using reflexive thematic analysis. Participant demographics were analysed descriptively. Synthesised member checking was used to enhance trustworthiness.

**Results:**

Sixty-five women from across Australia took part in the photovoice activity, attending virtual workshops and engaging in asynchronous online discussions that yielded 261 shared images and 899 comments. Thematic analysis generated seven themes: (1) BMI dictates care, (2) larger-bodied women feel excluded, (3) weight-centric care creates burdens, (4) stigmatising experiences leave lasting impacts, (5) women have embodied knowledge of their own health and wellbeing, (6) women hope for weight-inclusive care, and (7) individual maternity care providers can make a difference. Participants described weight-centric care as stigmatising, exclusionary, and emotionally burdensome, often undermining their autonomy and birthing confidence and leaving lasting impacts. Encounters with weight-inclusive care providers were described as empowering and healing. Women articulated a clear vision for respectful, individualised, and holistic maternity care.

**Conclusion:**

Larger-bodied women experience maternity care as weight-centric and stigmatising. Conversely, encounters with weight-inclusive providers contributed to positive care experiences, and shaped women’s vision for more respectful, strengths-based care. Larger-bodied women’s experiences inform the need for respectful, strengths-based care and informed choice. Findings will inform the co-development of national care guidelines to address weight stigma and improve maternity care for women in larger bodies.

## Background

Weight stigma is a widespread form of social stigma experienced by larger-bodied people, characterised by bias, discrimination, stereotyping and social exclusion [1]. Weight stigma is prevalent in healthcare interactions [2–4], harming larger-bodied people’s self-esteem, mental health, and quality of life, and contributing to their disengagement from health services, delays in seeking care, and mistrust of healthcare providers [4, 5]. Weight stigma has also been linked to behaviours that harm health, such as binge eating and exercise avoidance [6, 7].

As with other types of healthcare, those receiving maternity care are susceptible to weight stigma and its impacts, with weight stigma widely documented in women’s experiences and maternity care provider (MCP) attitudes. In Australia [3] and internationally [8, 9], women commonly report encountering weight stigma in their maternity care leaving them feeling judged, shamed, and guilty due to their weight. Likewise, there is a significant body of literature documenting stigmatising attitudes among MCPs [10–13]. Such attitudes contribute to less appointment time being devoted to rapport building and inquiries about health behaviours [14], with women experiencing care as focused narrowly on risks related to their body size [15]. Larger-bodied women may also be afforded less involvement in decision making about their maternity care [15], and less access to respectful maternity care [16].

This existing literature documents the nature and impact of weight stigma in maternity care but stops short of investigating solutions. Moreover, studies aimed at addressing weight stigma have rarely involved people with lived experience [17]. Only one other study has used codesign methods to develop resources to address weight stigma in maternity care [18], but focussed narrowly on antenatal care. Our study is part of larger program of research aiming to co-create comprehensive Best Practice Principles for Weight-Inclusive Maternity Care. Weight-inclusive approaches hold that individuals can optimise their health and well-being at any weight, if they have access to non-stigmatising health care [19]. Weight-inclusive healthcare stands in contrast to weight-centric approaches which regard body weight as a primary determinant of health, emphasising individual control over and responsibility for achieving and maintaining a “healthy weight.” [19]. Building on our successful pilot study [20], we conducted a strengths-based, participatory study with the aim of investigating larger-bodied women’s experiences of weight-centric and weight-inclusive maternity care. Throughout this paper, we strive to avoid stigmatising language, such as obesity, using terms preferred by our lived experience investigators, such as ‘larger-bodied women.’ We acknowledge that people of diverse gender identities, including transgender and non-binary people, also experience pregnancy and access maternity services. Our use of the word “woman” is intended in the sexed sense of the word and is not meant to exclude those who identify their gender differently. Similarly, terms like “maternity care” are used to maintain clarity, conciseness and accessibility to the broadest audience. We also use the term ‘maternity care’ expansively, intending to encompass childbirth education, fertility treatment, preconception care, antenatal care, intrapartum care, postnatal care, breastfeeding support and related allied healthcare.

## Methods

### Design

We adopted a consumer-led approach in which six lived experience investigators (AG, RT, TS, KB, LH, TC) worked alongside the academic team (BJ, LK, BH, JH, JAB, LC) to design and conduct a photovoice study to explore larger-bodied women’s experiences of maternity care. Photovoice is a qualitative, participatory research method where participants use images and narrative reflection to share their experiences, engage in critical dialogue, and promote social change [21, 22]. Our strengths-based approach recognised that larger-bodied women hold important knowledge about overcoming weight stigma and thriving before, during and after pregnancy. This approach allowed us to focus on what weight-inclusive maternity care does or could look like, and encompassed medical, midwifery, and allied health care across the preconception, antenatal, intrapartum, and postnatal periods. We also recognise that efforts to change weight stigma attitudes and beliefs should include empathy-evoking approaches [17]. Women’s narratives and visual images from this study may also play a critical role in helping MCPs consider larger-bodied women’s experiences more empathetically.

**Fig. 1:**
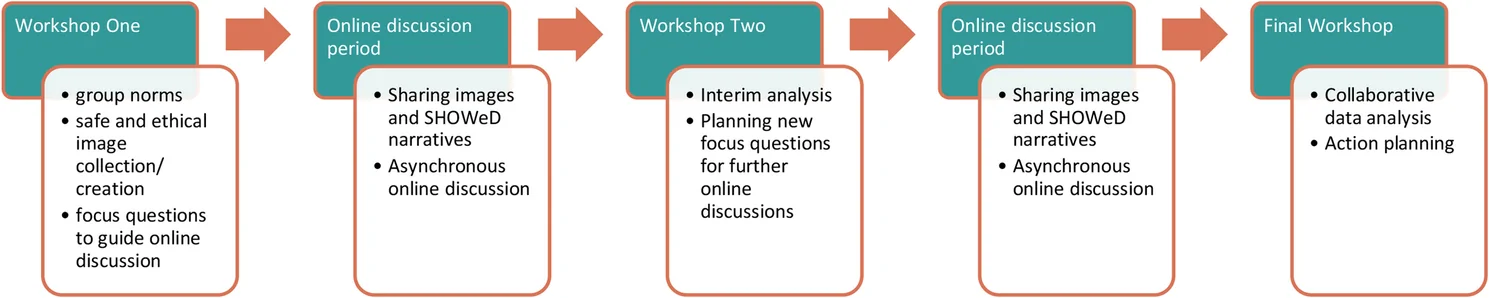
Photovoice process

Our photovoice study was conducted online to include participants from across Australia. Participants attended a series of 2-hour Zoom™ workshops, interspersed with periods of online photo sharing and discussion (see Fig. 1) in private groups on the study website. This study received ethics approval from the Human Research Ethics Committee at The University of Queensland (HREC Approval Number: 2023/HE001591).

### Participants, recruitment, and data collection

Eligible participants were aged 18 years or older, living in Australia, either currently or recently (within the last 3 years) pregnant, and perceived that their weight had impacted their maternity care. Participants also needed to have access to a suitable device to take photos and participate in online discussions and workshops. All participants provided written informed consent.

Information about the study was disseminated via paid social media advertising between December 2023 and February 2024. Participants were directed to the study website to review study information and, if interested, complete an online expression of interest via Qualtrics (Provo, UT), including eligibility and demographic questions. Members of the research team contacted each interested person by telephone to discuss the study, answer questions, and enrol them in an onboarding workshop. We anticipated that approximately 60 participants would deliver the necessary information power [23] to address the study’s aims, given the breadth of those aims, our emphasis on including women with diverse maternity care experiences, and the anticipated quality of online discussion co-facilitated by lived experience investigators. While this is a relatively large sample in the context of photovoice studies, it also enabled us to build an activated lived experience community, which we recognised as important preparation for championing future translation into practice [24].

**Fig. 2:**
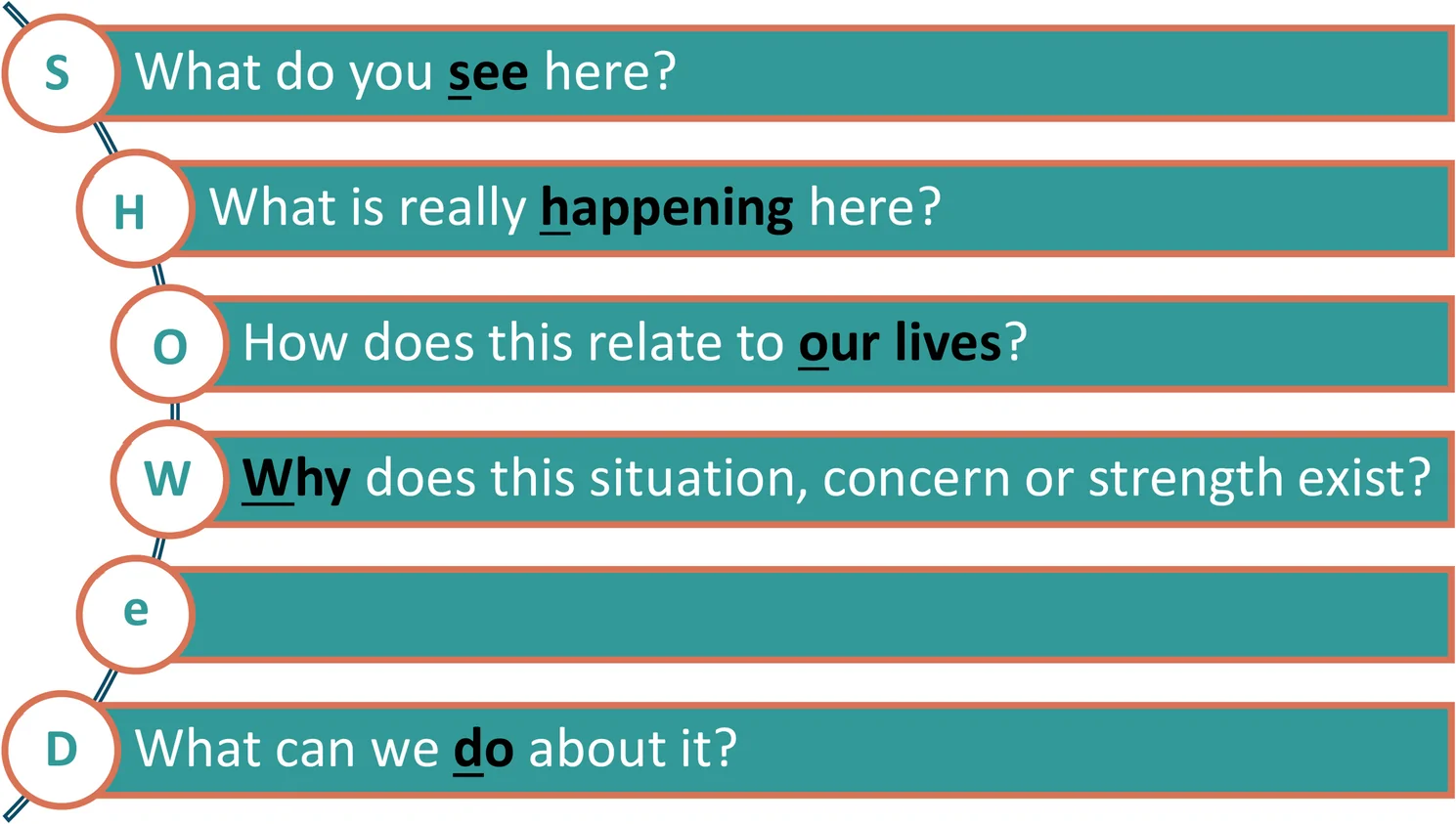
The SHOWeD Framework for Photovoice Analysis [21, 22]

In the initial onboarding workshops (February 2024), participants collaboratively agreed on group norms, reviewed the study aims, discussed safe and ethical photo collection/creation, and developed focus questions to guide photo sharing and online discussion (see Table 1). Participants were invited to share a diverse range of images, including existing photographs of themselves, photos they created to symbolise an aspect of their experience, AI-generated images and other visual artefacts they found online. Subsequent workshops in March and May 2024 engaged participants in a collaborative review of shared images, reflection on the patterns of shared experience, and action planning. Participants’ review of shared photos was guided by the SHOWeD framework (see Fig. 2). Workshops were facilitated by BJ (an experienced qualitative researcher with expertise in online group facilitation including on sensitive topics) and AG (a lived experience researcher) with input from other members of the lived experience and academic team.

**Table 1:** Questions Co-Developed with Participants

First sharing period
What does size inclusive care look and feel like?
How did I reclaim my agency in maternity care?
How did my past experiences shape my maternity care?
Second sharing period
How do different models of care differ in the way that they respond to and support larger-bodied women?
How does being told that you can’t birth in your preferred hospital or your preferred model of care impact your experience?
How well are larger-bodied women supported to feed their babies?
What knowledge and information would your past self have been grateful for?
How can we challenge the perception that “that’s just the way things are”?
Is there anything else that you would like to share?

Between each workshop, participants took part in private, online asynchronous discussion groups, sharing photographs, images or text to represent their experiences of maternity care. These private discussion groups were hosted on the study website, using the *EngagementHub* platform [25], and moderated by the study’s lived experience investigators. Participants could see, support, and comment on each other’s posts, similar to interaction on familiar social media platforms.

All workshops were recorded, and the Zoom™ transcript was checked for accuracy against the audio recording by AG. Identifiable comments (such as names or places) were replaced by pseudonyms. All photos and discussion threads were downloaded from the study website, into a Microsoft Excel spreadsheet, and similarly de-identified. Where individuals are identifiable in figures included in this paper, they have given specific written consent for the use of their image.

### Data analysis

Participant demographic data were analysed using descriptive statistics. Qualitative data (workshop transcripts, shared images, and online discussions) were analysed thematically using Braun and Clarke’s [26, 27] reflexive thematic analysis approach involving six stages: familiarisation with the data, generating initial codes, constructing themes, developing and reviewing themes, refining and naming themes, and writing up the findings. During the final workshop, participants identified patterns of shared meaning across the group’s experiences. These discussions were used as a starting point for three researchers (BJ, LK, AG) to independently code a selection of data using NVivo [28]. During analysis, these researchers met regularly to discuss progress, initially focussing on expansive coding rather than consensus. This process continued iteratively and recursively until related codes could be grouped into themes, which were labelled and defined. Other members of the research team reviewed a selection of the data against these themes, and BJ reviewed all data to check for cohesiveness within themes and search for disconfirming data.

To enhance trustworthiness, Synthesised Member Checking [29] was conducted. About six months after the final photovoice workshop, participants were recontacted via email, with a plain-language summary of the draft themes. Feedback, provided via an online survey, was incorporated into the data set and used to finalise the thematic map, which was agreed upon by the research team.

### Reflexivity

Reflexivity allows researchers to identify the background, values and assumptions that they bring to the research process, increasing rigour and transparency [30]. Our team is feminist, multidisciplinary and culturally diverse. Many of us identify as larger-bodied women. All of us are mothers, and recent maternity care experiences were especially salient for our lived experience authors. We recognise that our own intersecting identities have shaped our approach to this study, and in particular, our efforts to centralise lived experience, led by the first author (BJ) who is a maternity consumer-turned-researcher. Several of our team are current healthcare professionals, whose practice includes caring for larger-bodied pregnant women and whose research interests in the past have contributed to weight-centric maternity care. Throughout the research process we engaged in regular individual and group reflection, to surface assumptions, and test emerging ideas and decisions with the multidisciplinary team.

## Results

### Participant characteristics

Sixty-five people took part in the study (see Table 2), all of whom identified as women. Participants were diverse in terms of geography and age, and educational attainment was high. Participants were also diverse in terms of the maternity care they had accessed and their self-reported experiences and outcomes.

**Table 2:** Participant Demographics (*n* = 65)

	*n*	%
State of Residence		
Queensland (QLD)	22	33.6
Victoria (VIC)	19	29.2
New South Wales (NSW)	12	18.5
South Australia (SA)	4	6.2
Western Australia (WA)	5	7.7
Australian Capital Territory (ACT)	2	3.1
Tasmania (TAS)	1	1.5
Rurality (Monash Modified Model [31])		
Metropolitan (MMM1)	44	67.7
Regional (MMM2)	5	7.7
Rural (MMM3-5)	16	24.7
Age Group		
24–29 years	7	10.8
30–34 years	26	40.0
35–39 years	23	35.4
40–44 years	9	13.9
Diversity		
Aboriginal	3	4.6
Culturally and Linguistically Diverse^a^	3	4.6
LGBTQ + ^b^	5	7.7
Disability or chronic disease	7	10.8
Educational Attainment		
High School	7	10.8
Trade or Vocational Certificate	10	15.4
Bachelor’s Degree	26	40.0
Postgraduate Degree	22	33.9
Pregnancy Status^c^		
Currently pregnant	29	44.6
Pregnant within the last 3 years	57	87.7
Pregnant more than 3 years ago^d^	7	10.8
Number of Children		
No children	11	16.9
One	37	56.9
Two	11	16.9
Three or more	6	9.1
Types of Maternity Care Accessed^c^		
Childbirth education	44	67.7
Public hospital maternity care	51	78.5
Public midwifery-led care	19	29.2
Private midwifery care	10	15.4
Private obstetric care	17	26.2
General Practice care	54	83.1
Fertility care	17	26.2
Lactation consultant/breastfeeding support	40	61.5
Pre-pregnancy Experiences^c^		
Infertility or difficulty getting pregnant	25	38.5
Preconception healthcare or advice	30	46.2
Pregnancy Complications^c^		
Gestational diabetes	21	32.3
High blood pressure during pregnancy	14	21.5
Neonatal intensive (NICU) or Special Care Nursery admission	14	21.5
Another pregnancy complication	16	24.6
Pregnancy Losses^c^		
Early pregnancy loss or miscarriage	21	32.3
Termination of pregnancy	6	9.2
Birth Experiences^c^		
Vaginal birth	32	49.2
Caesarean birth	24	36.9
Water birth	2	3.1
Traumatic birth	20	30.8
Postnatal Experiences^c^		
Breastfeeding	46	70.8
Breastfeeding difficulties^e^	45	69.2
Postnatal depression or mental health challenges	32	49.2

^a^ Cultural and linguistic diversity can encompass a range of aspects including a person’s country of birth, their ancestry, where their parents were born, what language/s they speak, and their religious affiliation [32]

^b^ LGBTQ + is an umbrella term encompassing lesbian, gay, bisexual, transgender, and queer identities, with the “+” representing the many other diverse identities within these communities [33]

^c^ sums to > 65 participants, as participants were able to report multiple experiences in each category

^d^ participants were eligible to take part in the study because they had also been pregnant more recently (either currently, or within three years)

^e^ Participants who reported breastfeeding difficulties may or may not have identified as breastfeeding

### Thematic analysis

**Fig. 3:**
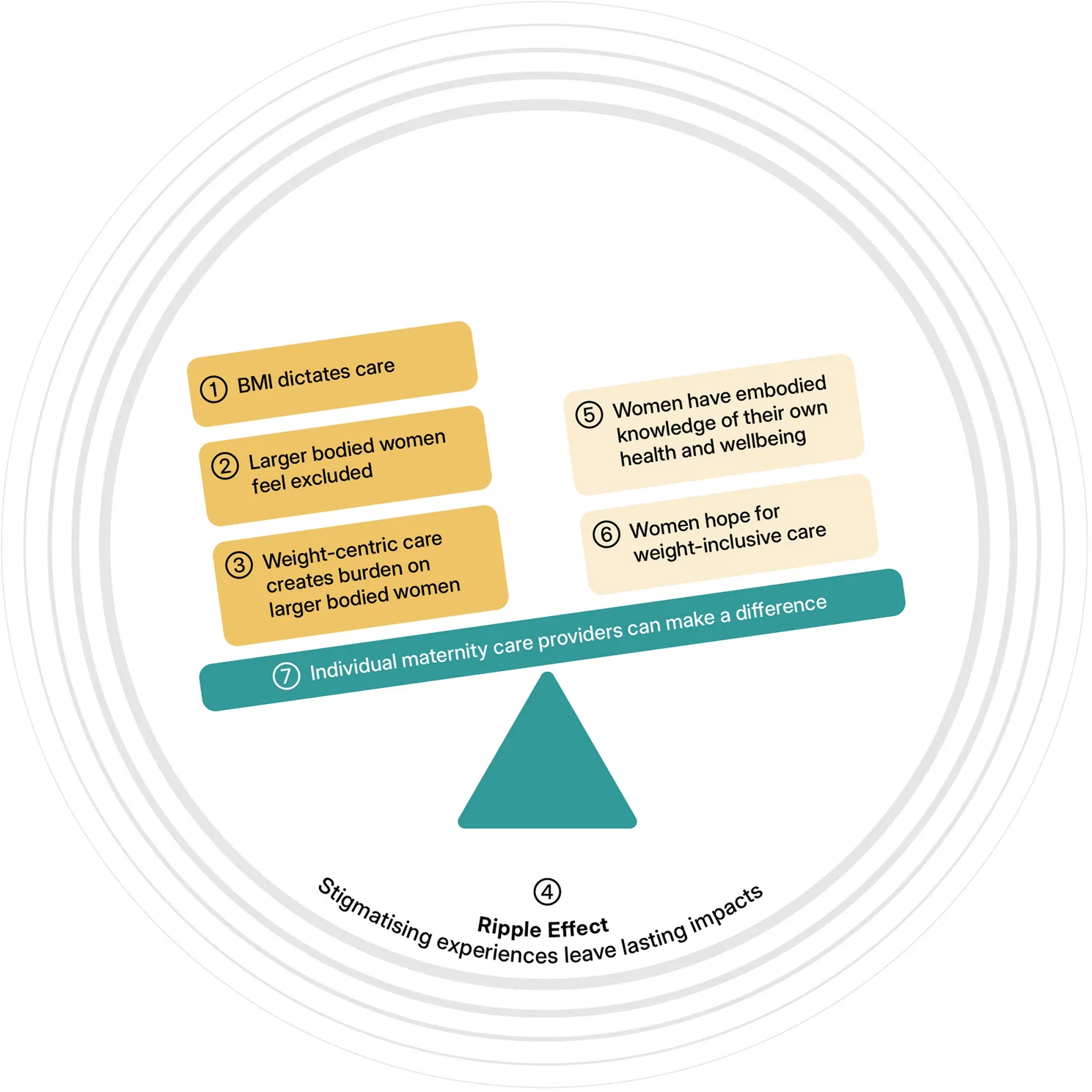
Thematic diagram

Participants shared 261 images in our private online discussion groups and made a total of 899 comments in the asynchronous online discussion threads on these images. From this data and the workshop transcripts, we generated seven themes to explore larger-bodied women’s experiences of maternity care (see Fig. 3). Themes 1–3, on the left of the figure, explore the negative and stigmatising experiences that women reported. The fourth theme surrounds all of the others, representing the enduring impact of stigmatising maternity care experiences. Conversely, on the right of the figure, two themes represent women’s positive experiences and hope for weight inclusive care. All of these themes ‘balance’ on the final theme ‘Individual maternity care providers can make a difference’, representing how participants’ experiences were shaped by interactions with individual MCPs. These themes are explored below, with illustrative quotations attributed to participants using pseudonyms. Where needed for clarity or brevity, words have been added (denoted by [square brackets]) or omitted (denoted by …). Reported speech is indicated by “quotation marks”.

####  BMI dictates care

The first theme, ‘*BMI dictates care’*, encapsulates how many women perceived that their BMI was the only aspect of their health and wellbeing considered when MCPs made decisions or recommendations about their care.

**Fig. 4:**
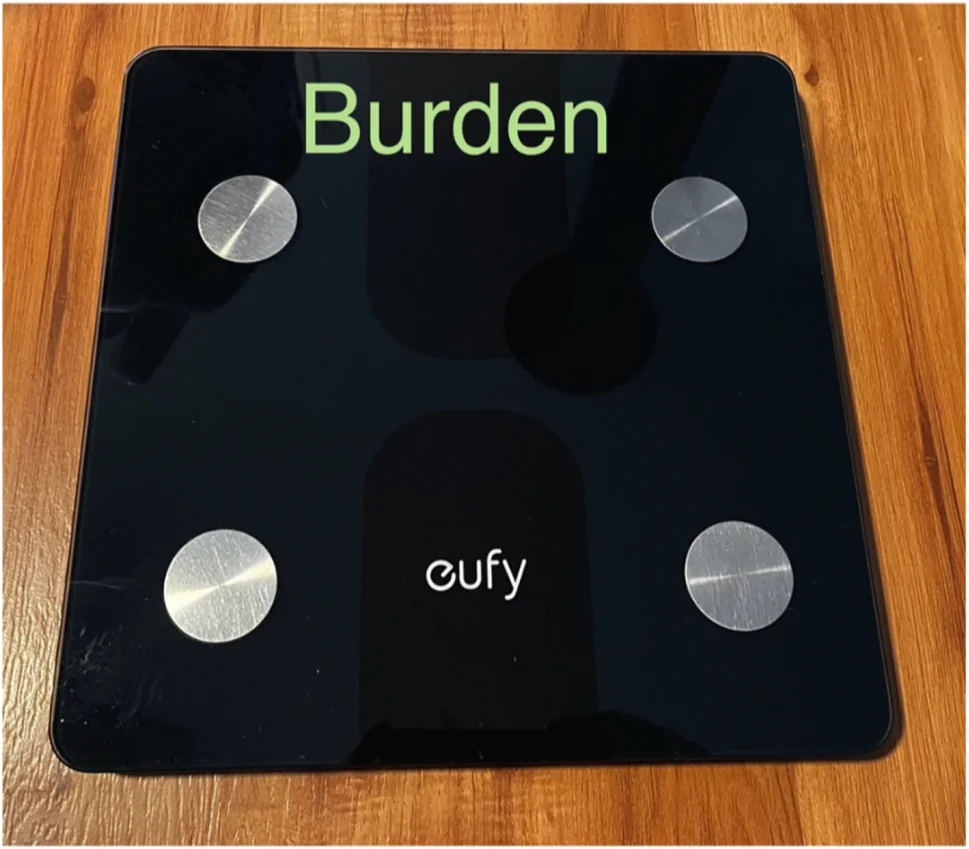
Image shared by Lisa (30–34 years, Metropolitan, with permission)

*My weight was the focus of every single appointment. I was weighed and lectured if I put on any weight.* (Lisa, 30–34 years, Metropolitan, with Fig. 4)

Despite MCPs often reportedly acknowledging that BMI was a faulty indicator of health, women still felt that their BMI and weight were used to limit their birthing options.

*I wanted a natural birth and was strongly advised against it. My weight was the deciding factor and I was told statistically due to my weight I would struggle to birth naturally and require either instrumental assistance or an emergency caesarean section … I feel I was robbed of an experience.* (Ashley, 35–39 years, Metropolitan).

Weight-centric approaches made women feel like their health was defined by this single number, with their weight blamed for a diverse array of experiences, including fertility issues and pregnancy losses as well as pregnancy, labour and birth complications.

*Any pregnancy related complications were almost always related back to my weight but in a way where I felt like I was at fault when often these complications are just really bad luck … hyperemesis gravidarum*,* gestational diabetes*,* polyhydramnios.* (Katie, 30–34 years, Rural)

*I have peers that struggled to conceive and their preconception appointments were full of empathy*,* support and referrals to IVF clinics*,* acupuncture clinics and fertility support agencies. In my appointments the only topic of conversation was my weight and BMI. My referrals were to nutritionists*. (Holly, 30–34 years, Metropolitan)

In other cases, women perceived that their concerns have been dismissed because of their weight, directly contributing to poor quality care and complications.

*I was brushed off by midwives at first telling me that I couldn’t feel the baby [moving] due to me being obese. I … was told that I was fine without being properly assessed … My son was resuscitated and intubated at birth and was in the NICU for 10 days … I really believe that it was prejudice about my weight that resulted in my baby being so unwell.* (Riley, 30–34 years, Metropolitan)

In some cases, women had been denied access to desired healthcare (such as fertility care) until they lost weight.

*“Lose weight*,* that’ll solve your issues.” What issues? Every test came back within range. I was in perfect health except for that silly BMI number. Why was I denied any help because of an outdated weight scale?* (Morgan, 30–34 years, Metropolitan)

Many women were met with what they perceived to be arbitrary rules and felt that their choices were limited by policies requiring extra monitoring and interventions, or excluding them from specific models of care, or entire hospitals.

*It [BMI] determined which hospital I birthed in*,* whether I could access a water birth*,* how many GD [gestational diabetes] tests I had*,* and whether I needed to take aspirin throughout my pregnancy to ward off preeclampsia.* (Holly, 30–34 years, Metropolitan)

*[I was]… suddenly told “we’re doing the sweep today”. When I refused the sudden plan I was told “you’re drastically increasing the risk your baby doesn’t survive and it won’t be the hospital’s fault”. I wasn’t even 37 weeks at that time. But that was shocking to my mental health and made me very stressed and panicked - not something you should be in the later stages of pregnancy.* (Kelly, 30–34 years old, Metropolitan)

Women reported that this (over)emphasis on BMI undermined the woman-centredness of their care. They wanted explanations about why certain policies were in place and felt dismissed by explanations that did not extend beyond “it’s policy” or “because your BMI is too high” (Savannah, 24–29 years, Metropolitan). Women also received contradicting information from MCPs and felt that decisions about their care had been based on what they perceived to be insignificant differences.

*I was only one point on the BMI scale or a couple of kgs [kilograms] over the ‘cut off’ to birth at my preferred hospital.* (Cynthia, 35–39 years, Rural)

#### Larger-bodied women feel excluded

The second theme, ‘*Larger-boded women feel excluded*’, explores how women perceived that maternity care environments and practices did not accommodate them. This included exclusion from preferred models of care, often based on weight-centric risk assessments, which women experienced as punitive.

*I always get so angry when I hear about ‘choice’ when referring to models of care… I was taken into a room and told I had been moved to the Obstetric model of care as I never ‘qualified’ for the midwifery program. I was crushed… I felt robbed. I felt like I was being punished. My choice had been taken away. It sucked the joy out of the rest of my pregnancy.* (Jade, 30–34 years, Regional)

Women also felt excluded when they encountered healthcare environments and attitudes that catered only to smaller bodies. These experiences often stemmed from inadequate equipment, such as waiting room chairs, blood pressure cuffs or fetal heart monitors. Women were often aware that inadequate equipment could have significant implications for the safety and quality of their care.

*This [ill-fitting blood pressure cuff] was constantly an issue for me in both of my pregnancies. They would only ever have the “standard” sized cuff available which would give wildly inaccurate readings. As soon as they found the next size up perfect blood pressure! The difference it would make in having that available at the start would be amazing!* (Tammy, 30–34 years, Rural)

Women also felt excluded by the lack of representation of larger-bodied women in maternity care information, noting that positive images of larger-bodied pregnant women were rare.

**Fig. 5:**
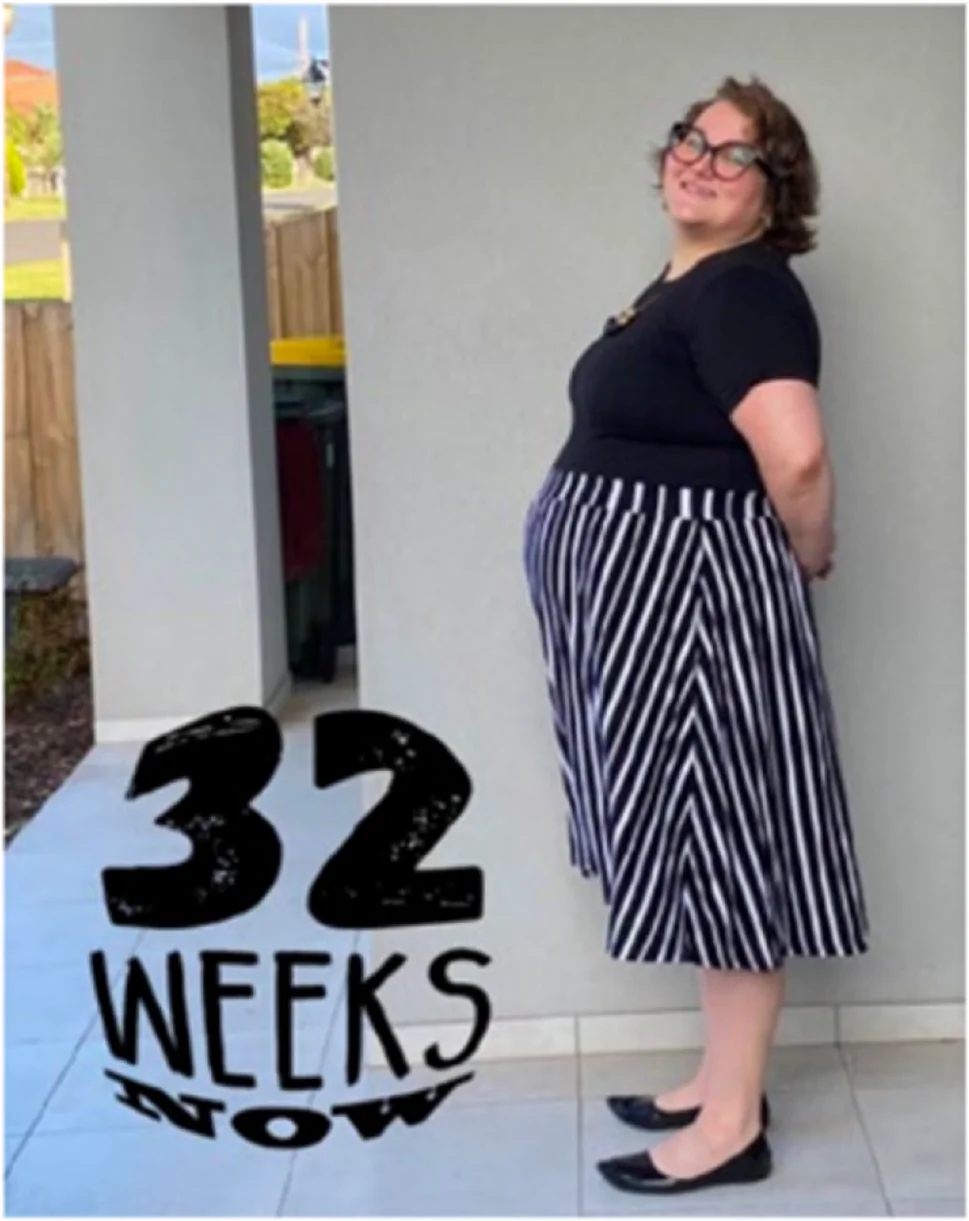
Image shared by Kristin (35–39 years, Rural, with permission)

*All the pictures we see of pregnant women tend to have round bellies … This is reiterated in medical settings where the images of pregnant women are often not of bigger bodies. There’s a lot to be said for diverse imagery to validate a diverse range of normal. I’d love to see a range of pregnant bellies in artwork or even in promotions/marketing materials for hospitals.* (Kristen, 35–39 years, Rural, with Fig. 5).

Many women had looked forward to pregnancy experiences such as enjoying their pregnant bodies and breastfeeding, but instead felt stigmatised and excluded.

*I wish I could have had the same experiences as my straight-sized peers did… without being made to feel separate or other.* (Holly, 30–34 years, Metropolitan).

In a particularly powerful image of feeling othered and unwelcome, “like a burden or a problem” (Riley, 30–34 years, Metropolitan), one woman articulated this as “feeling like a blob fish” during her maternity care.

**Fig. 6:**
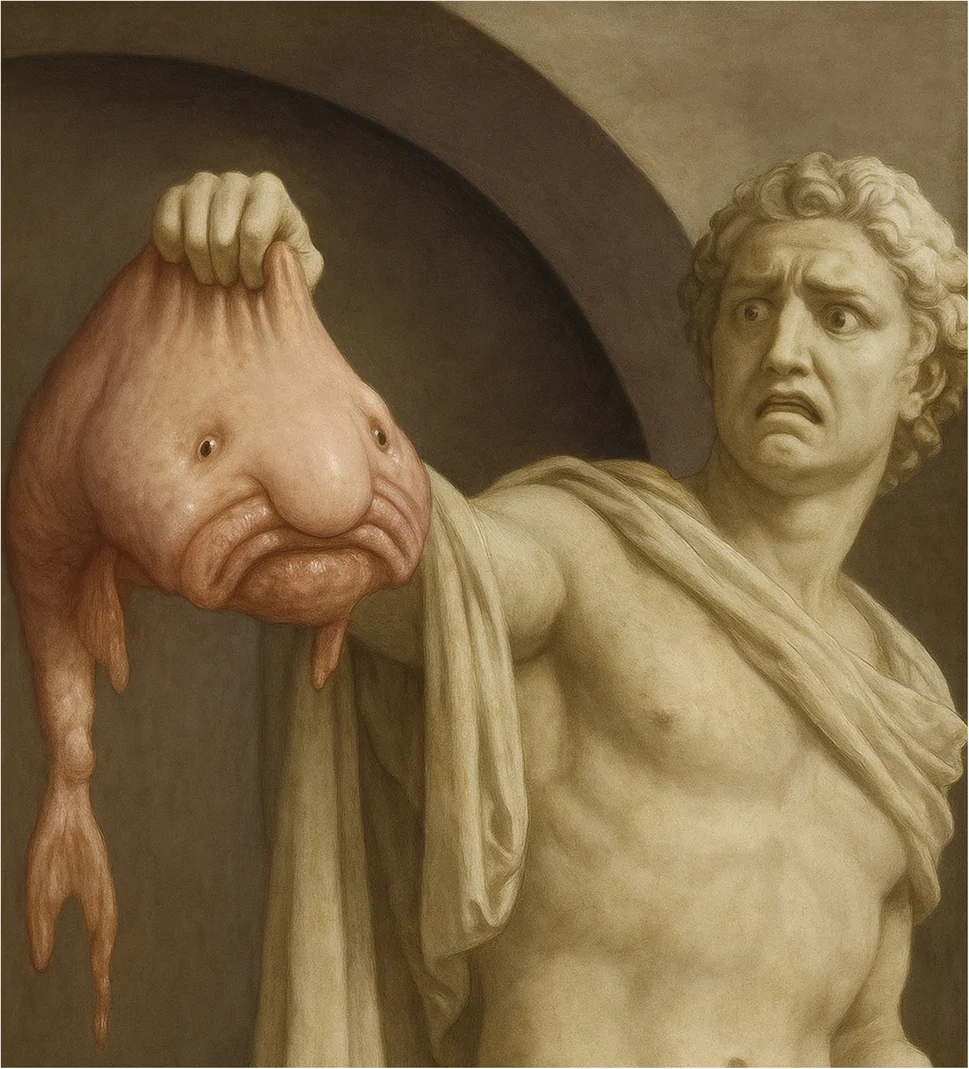
AI generated image shared by Claudia (30–34 years, Metropolitan, with permission)

*I was looking for inspiration for a picture and all I could think about was feeling like a blob fish. The fear on a practitioner’s face when looking at me. Everything was fearful.* (Claudia, 30–34 years, Metropolitan, with Fig. 6)

#### Weight-centric care creates burdens on larger-bodied women

In the third theme, ‘*Weight-centric care creates burdens on larger-bodied wome*n’, participants described how weight-centric maternity care had imposed additional physical and emotional burdens on them while they were trying to conceive, as well as during pregnancy, birth, and postpartum. This burden often arose from additional appointments. Women experienced these additional appointments as a presumption of pathology associated with their weight.

*You’re either the right size or the wrong size and if you’re the wrong size you have to go through so many extra steps - get the gestational diabetes test twice (the first to test for underlying diabetes). See the anaesthetist (to make sure they can access my back and my windpipe is clear then tell me the black and white number I can’t go over). See the OB at the hospital (to confirm they are happy to have me and tell me when they need to induce because of my weight). Then extra ultrasounds at 32 and 36 weeks because “the fundal measurement is never accurate for a women of your BMI”.* (Jamie, 20–24 years, Metropolitan)

In some cases, women accepted being classified as ‘high risk’, but noted that this added significant emotional burden and fear about the impact their weight might have on their unborn baby.

**Fig. 7:**
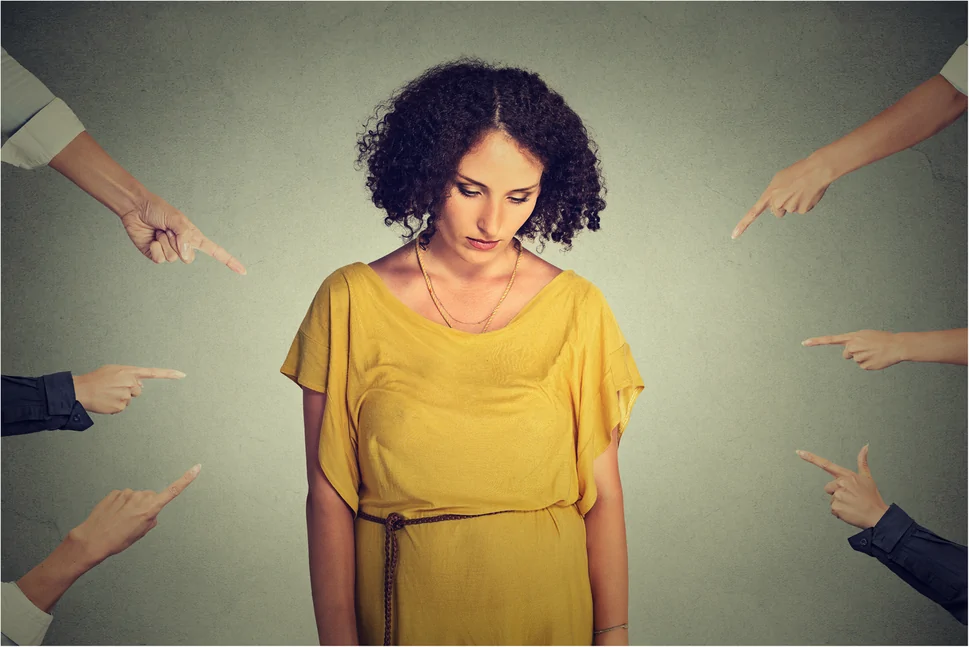
Image shared by Renata (35–39 years, Metropolitan, from Shutterstock)

*I felt judged by those caring for me … I was constantly made to feel bad for being on the bigger side with my size and weight and was often made to feel scared of what being overweight could do to my unborn child.* (Renata, 35–39 years, Metropolitan, with Fig. 7)

In other cases, the ‘high risk’ label did not accord with women’s own sense of their health and wellbeing. Nonetheless, many felt compelled to accept additional tests in an effort to validate their health status. When tests came back normal, women perceived that MCPs distrusted the results and re-ran tests, further burdening women.

*“Oh your bloods came back perfect. Must be wrong. Let’s do it again” or “Oh your second gestational diabetes test came back perfect. Must be wrong. Let’s do it again.”* (Claudia, 30–34 years, Metropolitan).

Women perceived that MCPs made assumptions about their health and the inevitability of complications and interventions. Women experienced this as a lack of belief in their ability to conceive, or to have a uncomplicated pregnancy and birth.

*When I was in the process of trying to conceive. It took me 6 months of trying … I saw a doctor about my fertility who every time I’d visit she’d be like “Have you tried a Mediterranean diet?”* (Stacey, 35–39 years, Metropolitan).

*[There were] very early conversations about large babies and needing an induction at 39 weeks while others were trusted to be able to carry and deliver their babies in their own time.* (Holly, 30–34 years, Metropolitan)

In the context of curtailed choices and MCPs who saw intervention as inevitable, women carried the burden of needing to advocate strongly for themselves. One woman described this need for self-advocacy as arising from a sense that she was *“treated as though I was guilty until proven innocent*,* or in my case*,* unhealthy until proven healthy”* (Gabrielle, 30–34 years, Metropolitan). The need for self-advocacy led many to take on additional research and mentally prepare for appointments.

*I need to do lots of work so that I am treated like a regular size*,* pregnant person. There’s a lot of extra work that I feel like I have to do just to get a basic level of care… You’re going in for a fight*,* even if you don’t want to*,* you have to be ready to argue your point of view and fight for the birth that you want*. (Esther, 30–34 years, Metropolitan)

For some women, this research included their prior knowledge of the MCPs at their hospital and they often used combative metaphors to describe these encounters.

*I had my last three babies at the same public hospital and had worked out my own take on each doctor. I used to check with my midwife which OB [obstetrician] would be on a week or two in advance so I could mentally prepare for another ‘round’ with the doctors.* (Tracy, 35–39 years, rural)

Women also reported financial and logistical burdens arising from weight stigma. In some cases, these financial burdens related to travelling longer distances to access care or taking time off work and paying for parking to attend duplicate medical appointments or undertake additional tests.

*The receptionist told me [that the nearby community health centre] wasn’t operational. It was. Just not for higher BMI women. So*,* I had to drive further*,* I had to pay a ridiculous amount for parking*,* I had to walk a long way because car parks are sparse at [the] hospital*,* I had to wait hours for my appointment. I also had to organise a babysitter because I wasn’t able to have my son in daycare like I would for antenatal appointments if they were close to home. Just making a hard situation even harder… All because I was high risk just for being in a larger body.* (Chloe, 24–29 years, Rural)

Some women also engaged doulas, private midwives or sought out weight-inclusive obstetricians/general practitioners to support them in advocating for themselves. While valued, this support was also an additional financial burden.

*…A private midwife in my local area*,* who is now caring for me in my second pregnancy … has been amazing and supportive throughout this journey*,* and not once has my weight been a question. Although I will still birth at the local public hospital*,* she will be there to help me and my husband advocate for the care we deserve.* (Jade, 30–34 years, Regional)

#### Stigmatising experiences leave lasting impacts

The fourth theme, *‘Stigmatising experiences leave lasting impacts’*, captures the ongoing impact of stigmatising experiences that women encountered during their maternity care. Women reported lasting impacts relating to traumatic births, mental health, and disengagement from health care. In some cases, women linked weight-centric care with traumatic birth experiences which often stemmed from communication that women perceived as fearmongering. This had a lasting impact on their wellbeing.

*She told me that I had to be induced because I was overweight*,* and my baby might die if I didn’t. The induction led to further complications during labour*,* the effects of which are still with me years later.* (Kylie, 30–34 years, Metropolitan)

Many women had wanted relationship-based midwifery care, believing that this would result in more positive birth experiences and a better chance of getting their needs met. Exclusion from these valued and evidence-based models of care had lasting impacts for women.

**Fig. 8:**
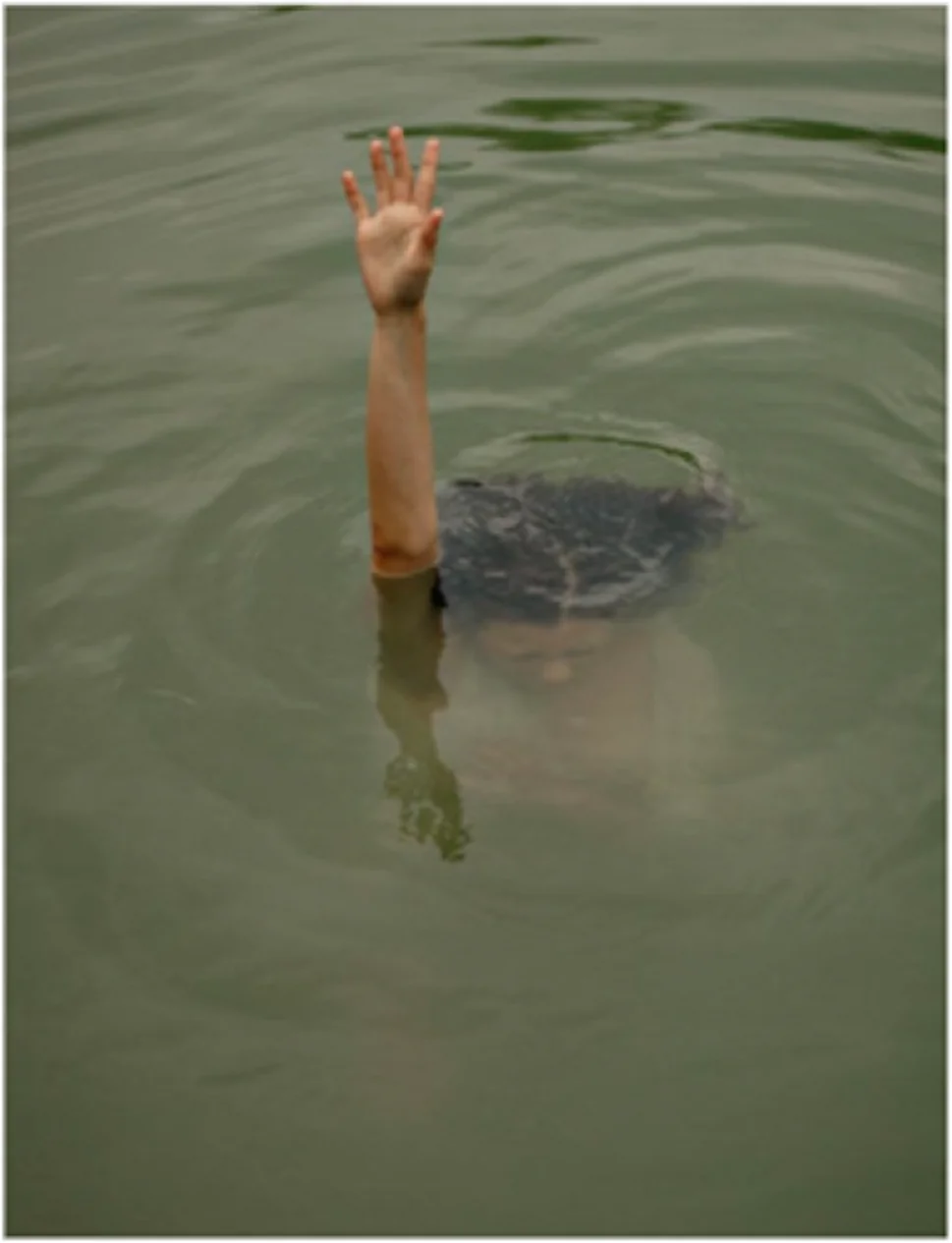
Image shared by Regina (35–39 years, Metropolitan, with permission)

*I chose this image to represent a feeling of not being heard or being left to drown. I truly feel if I’d had access to better relationship-based care with a trusted circle of known midwives … I would have much more psychological and emotional safety going into delivery and a better chance of those care providers being attuned to my needs and wishes during birth*,* preventing unnecessary trauma.* (Regina, 35 − 29 years, Metropolitan, with Fig. 8)

Other women experienced adverse mental health outcomes, including post-traumatic stress symptoms, anxiety and depression. In some cases, this had resulted in self-isolation or needing to take medication, highlighting the severity of their conditions.

*Being told I wasn’t allowed to birth in my local hospital … purely because of my BMI … Every appointment … filled me with anger … It made me sad but also incredibly anxious. Fearful of what was going to happen … my anxiety has peaked and due to the whole experience I have had to be back on anti-depressants.* (Cynthia, 35–39 years, Rural)

For some women, weight-centric maternity care had triggered disordered eating patterns. Women often linked these experiences to being directed to closely monitor their weight during pregnancy.

*I was convinced the pregnancy wouldn’t stick and had obviously internalised the previous ‘advice’ that my [pregnancy] losses were due to my size … I complied with every request*,* recommendation and demand made of me by the medical system. I became unhealthily fixated on what I ate*,* what I did*,* and how I did things. I weighed myself regularly*,* tracked calories*,* and counted macros to the point [that] I was mentally distressed most of the time.* (Kristen, 35–39 years, Rural)

In other cases, concerns about weight stigma led women to limit their contact with maternity services, and in some cases, disengage from mainstream maternity care entirely.

**Fig. 9:**
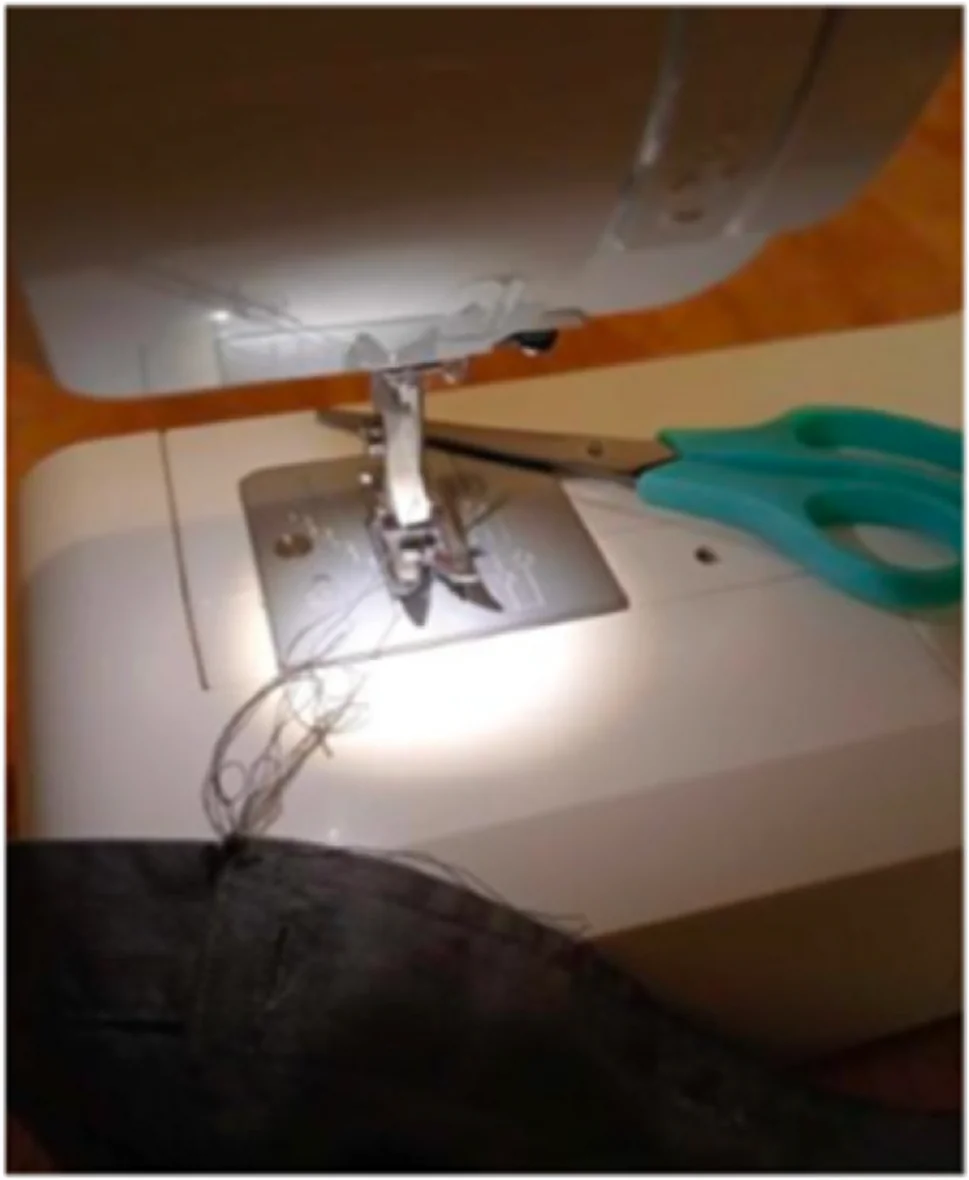
Image shared by Tracy, 35–39 years, Rural, with permission)

*I took this photo to symbolise the frustration I had being caught up in a health system that offered low risk women high risk care. These past experiences led to my delaying/avoiding doctors’ appointments where possible*,* delaying notification of early labour signs*,* delaying the time I would turn up to the hospital in labour*,* sometimes under-reporting symptoms during appointments*,* and dietary alterations/exercise to reduce BMI.* (Tracy, 35–39 years, Rural, with Fig. 9)

#### Women have embodied knowledge of their own health and wellbeing

The fifth theme, *‘Women have embodied knowledge of their own health and wellbeing’* was a counterbalance to the negative experiences that dominated larger-bodied women’s maternity care and was identified as a source of strength by participants. Many women experienced pregnancy, birth, and parenting as a time of positive transformation of their body image.

**Fig. 10:**
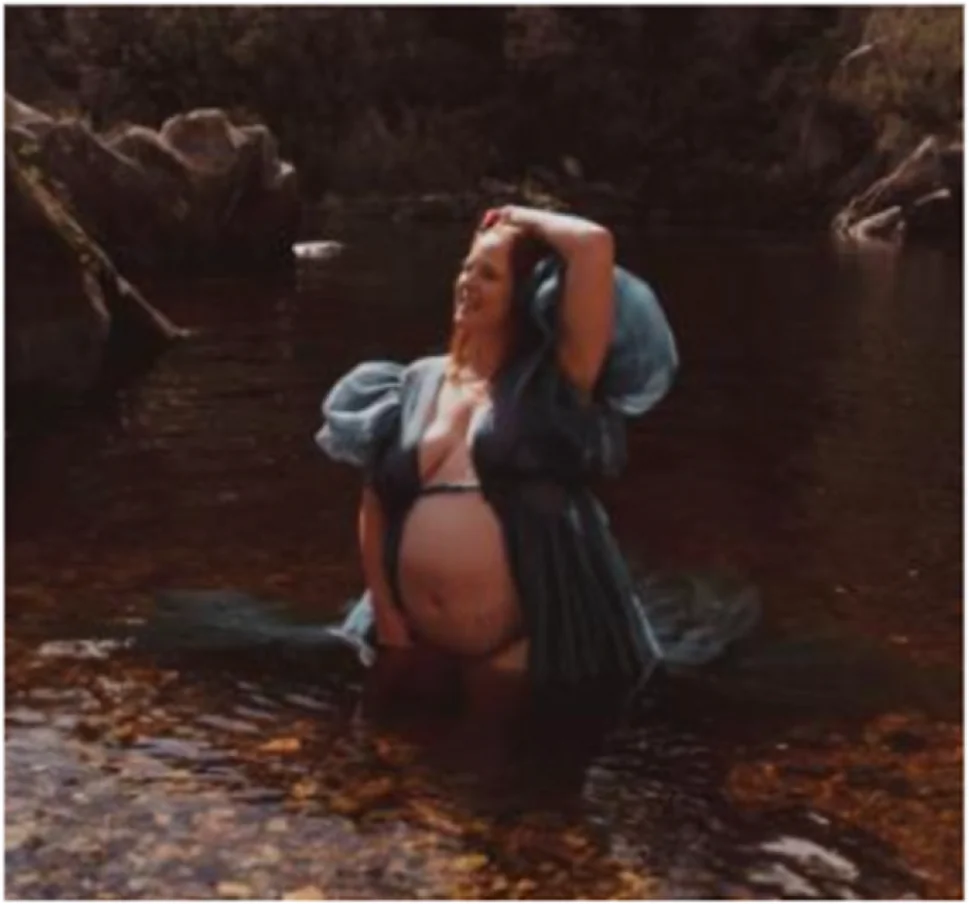
Image shared by Jamie (30–34 years, Metropolitan, with permission)

Honestly, deep down I had been feeling incredible about myself. It was the best I had ever felt and pregnancy had been the greatest thing for me. I was just made to feel like I couldn’t feel this way. (Jamie, 20–24 years, Metropolitan, with Fig. 10)

*Pregnancy evoked complex emotions around my sense of body image. While lack of control over my weight was at times distressing*,* late pregnancy was the only time in my life where I’ve loved the way my body looked.* (Regina, 35–39 years, Metropolitan)

Although their embodied knowledge of their own health and wellbeing was challenged by stigmatising maternity care, it was often strengthened by seeing their babies thriving.

**Fig. 11:**
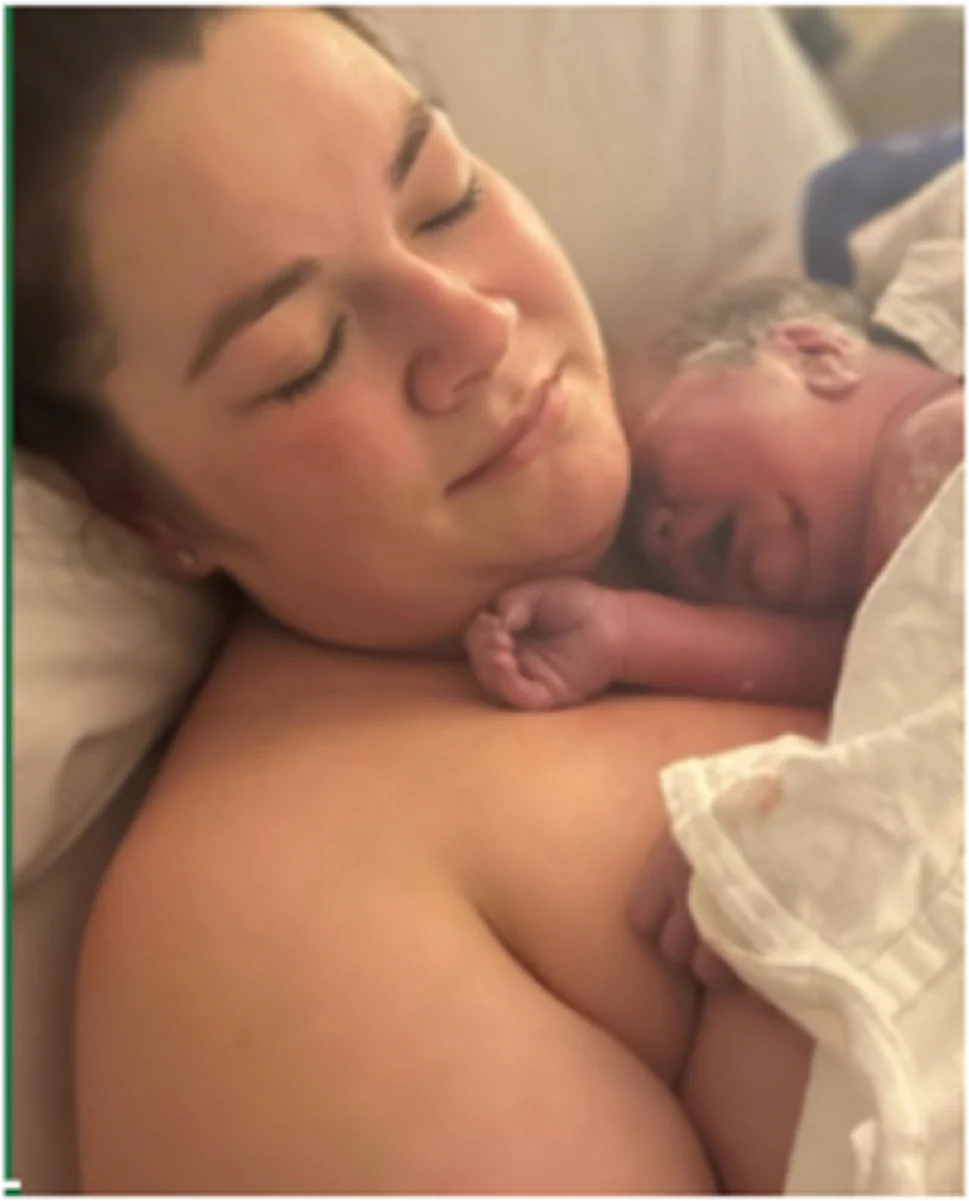
Image shared by Holly (30–34 years, Metropolitan, with permission)

*Since having my son*,* I have flourished and thrived as a mother. There were times in my pregnancy when I felt like an imposter who was undeserving of this experience. I have proven myself worthy of being a mum and that I am good at this!* (Holly, 30–34 years, Metropolitan, with Fig. 11)

#### Women hope for weight-inclusive care

The sixth theme, ‘*Women hope for weight-inclusive care’* captured women’s clear vision for how maternity care could be, often inspired by encounters with weight-inclusive MCPs. Women defined weight-inclusive care as individualised, holistic, and available to everyone.

**Fig. 12:**
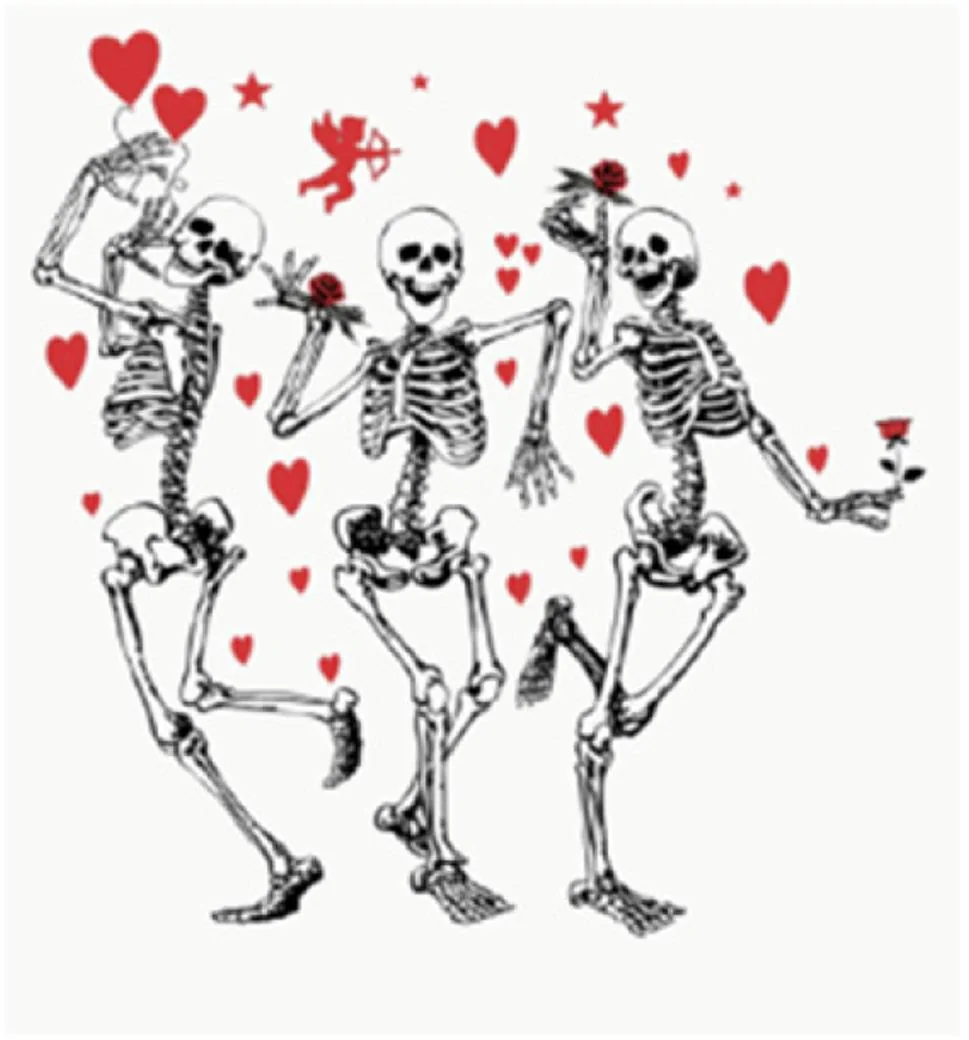
Image shared by Celeste (35–39 years, Metropolitan, From Etsy artist ArtSvgTR, with permission)

*Weight-inclusive care really for me means being treated with respect*,* patience*,* love*,* and understanding… each patient is unique and needs some accommodations… we should be able to offer holistic care that respectfully accounts for our differences and moves on treating each as a human being … We’re all the same underneath and that’s how we should treat each other… with excitement for their journey*,* compassion for their challenges.* (Celeste, 35–39 years, Metropolitan, with Fig. 12)

Women wanted MCPs to undertake more training in compassionate communication, especially around sensitive topics, such as weight and risk. They also wanted MCP education to strategically include diverse body shapes and sizes, and foreground the need for equity, dignity and respect.

*I think it starts with education in university. Maybe they need to have larger-bodied people present to university students to educate them on what health at any size looks like and how their actions and words can impact their therapeutic relationship with patients.* (Ruth, 35 − 29 years, Rural)

Women believed that compassionate communication could extend to more balanced conversations about risk and greater acknowledgement of positive outcomes, with less focus on their weight.

*They accepted a guesstimate [of weight] and never asked again. No talk of “big babies”*,* no talk of induction or caesarean unless medically necessary and no talk of an epidural. All conversations were to be positive and lead back to “my body can do this”. … [As I] was able to build a rapport with the OB it becomes clear that everything I wanted he also wanted for me as well so that made it a lot easier.* (Sydney, 24–29 years, Metropolitan)

Women appreciated explanations that health outcomes could be due to a range of factors, not just their weight.

*I was enjoying GP shared care where it wasn’t about numbers*,* statistics*,* or weight; instead it was about me and my baby overall and how we were progressing.* (Janet, 35–39 years, Regional)

#### Individual maternity care providers can make a difference

The final theme, ‘*Individual maternity care providers can make a difference*’ captured women’s experience that the difference between positive and negative maternity care experiences often came down to a single provider or interaction. All participants in this study reported stigmatising encounters, but many had also experienced respectful, compassionate care. Women recounted feeling supported by providers who took the time to understand their personal needs, helping them to regain courage, control, and confidence in their bodies and decisions.

**Fig. 13:**
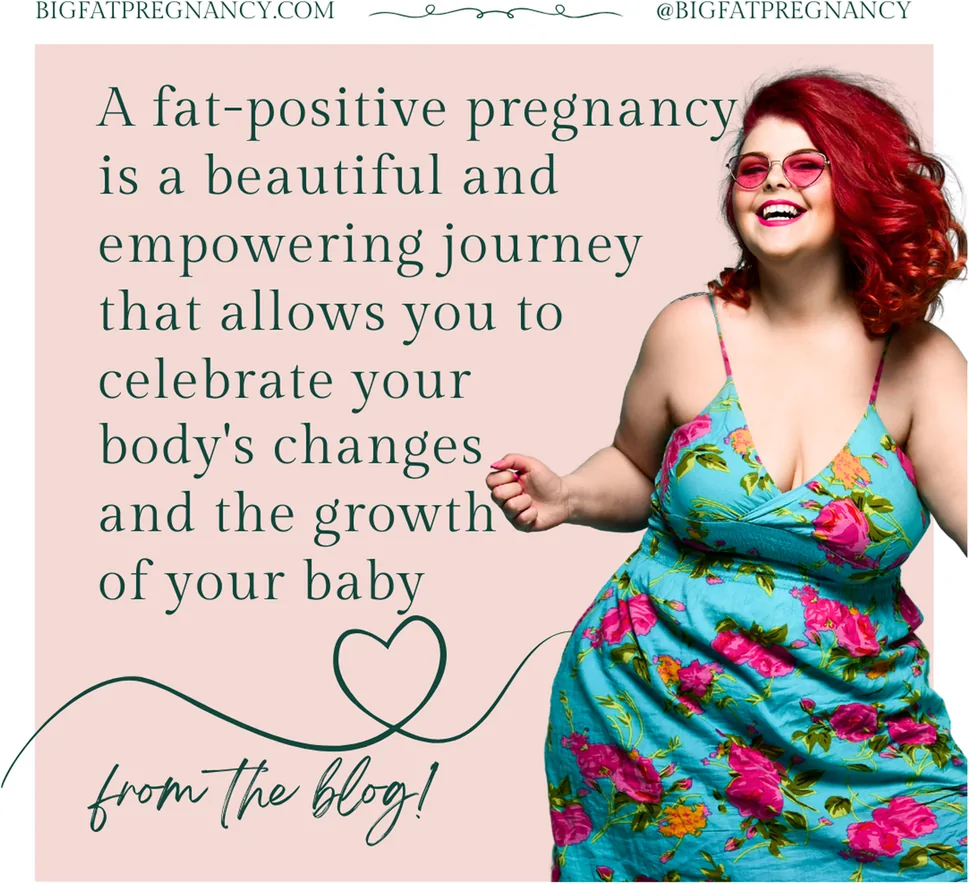
Image shared by Lori (35–39 years, Metropolitan), from Instagram handle BigFatPregnancy, with permission

*They saw me as a person and listened to each question and challenge I raised. I have been more empowered to be persistent and ask questions to have a sense of agency through my pregnancy.* (Lori, 35–39 years, Metropolitan, with Fig. 13)

In the context of weight-centric maternity care, women noted that individual MCPs who provided weight-inclusive care stood out and made a significant positive impact.

**Fig. 14:**
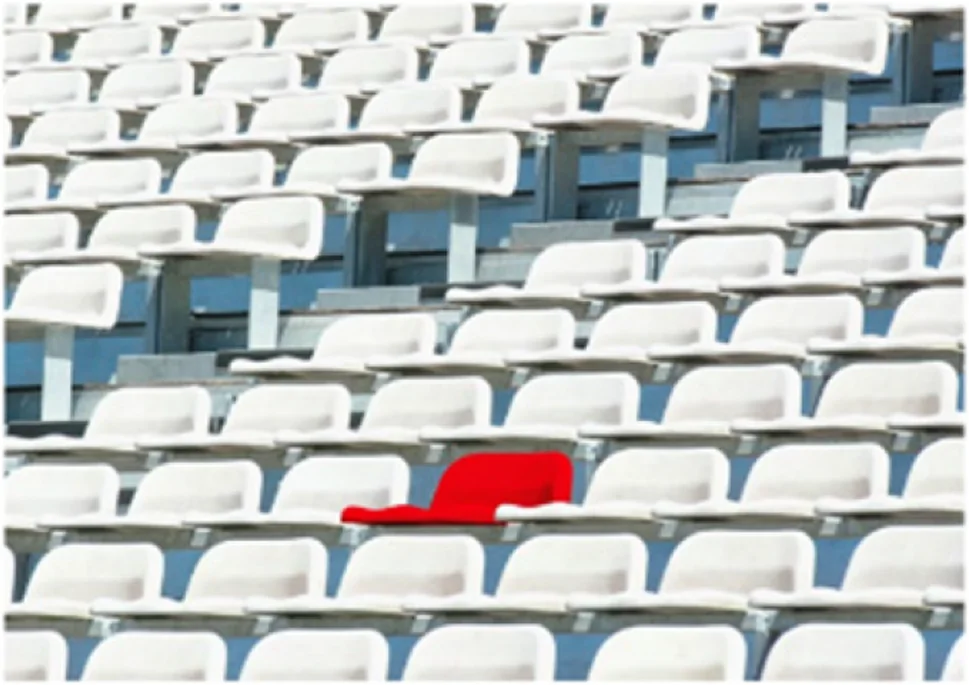
Image shared by Regina (35–39 years, Metropolitan, adapted from Jan van der Wolf/Pexels)

*After being seen by a revolving door of doctors who were very clinical and impassive in their judgement of what was best for me*,* this one clinician really stood out with above all their compassion*,* but also how they made the effort to give objective advice … She truly engaged with me as an individual*,* asked how I was feeling about my care … affirming that my mental health and choice were more important than a risk-averse hospital policy pressuring me to be induced at 38 weeks for no other reason than my BMI. She gave me back a sense of control that I really needed at the time.* (Regina, 35–39 years, Metropolitan, with Fig. 14)

In some cases, women described this kind of supportive, respectful care as healing.

**Fig. 15:**
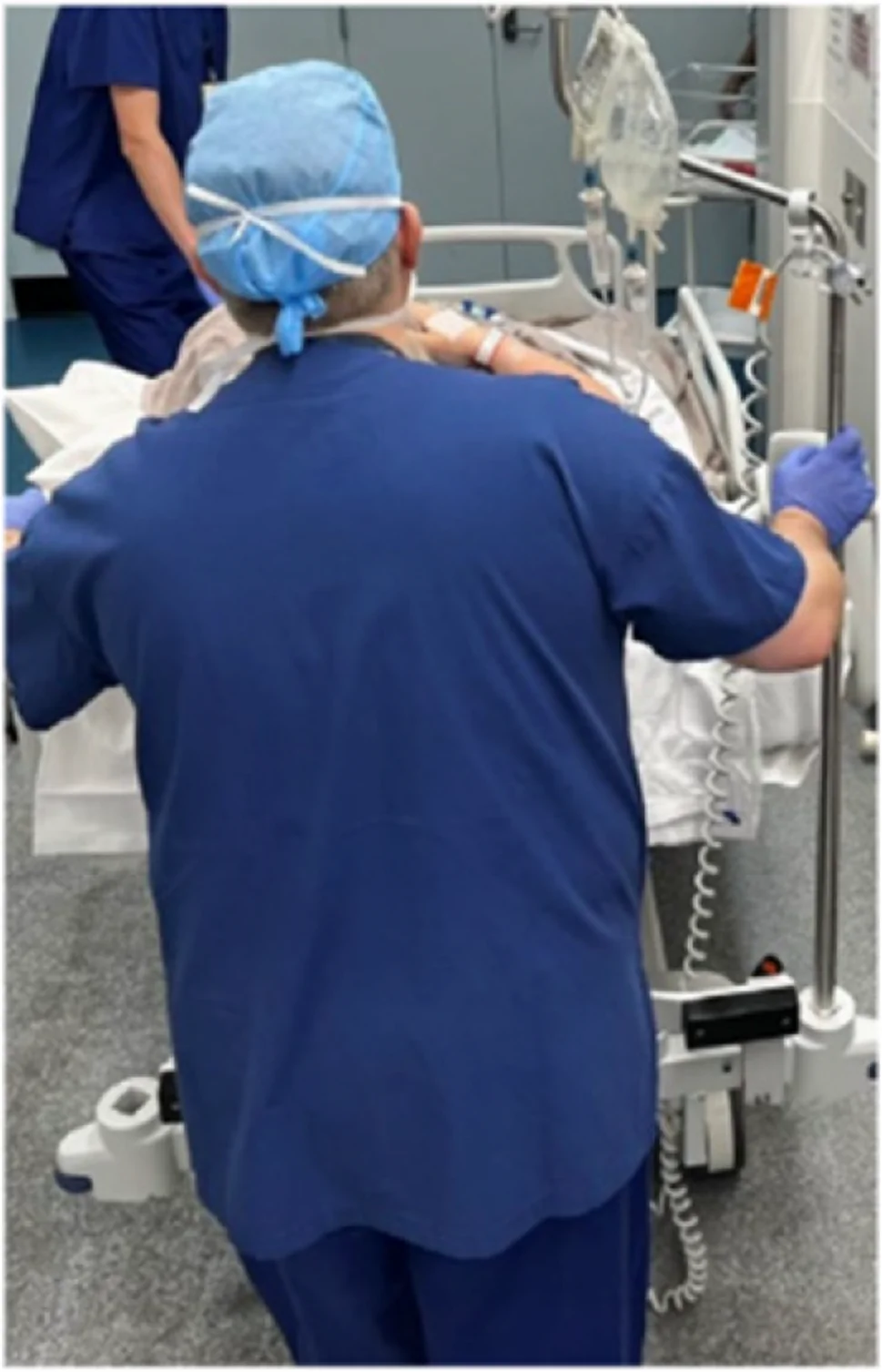
Image shared by Riley (30–34 years, Metropolitan, with permission)

*This was my anaesthetist pushing my bed to recovery after my caesarean section … I have never felt so cared for in a medical setting… I usually feel judged by medical professionals because of my weight … it was the first time I felt like a normal-sized person as my weight was not mentioned or made an issue. I wish that everyone could experience what I did*,* it was incredibly healing.* (Riley, 30–34 years, Metropolitan, with Fig. 15)

Conversely, MCPs perceived as dismissive, judgmental, blunt, or aggressive left lasting negative impacts on women’s healthcare experiences.

**Fig. 16:**
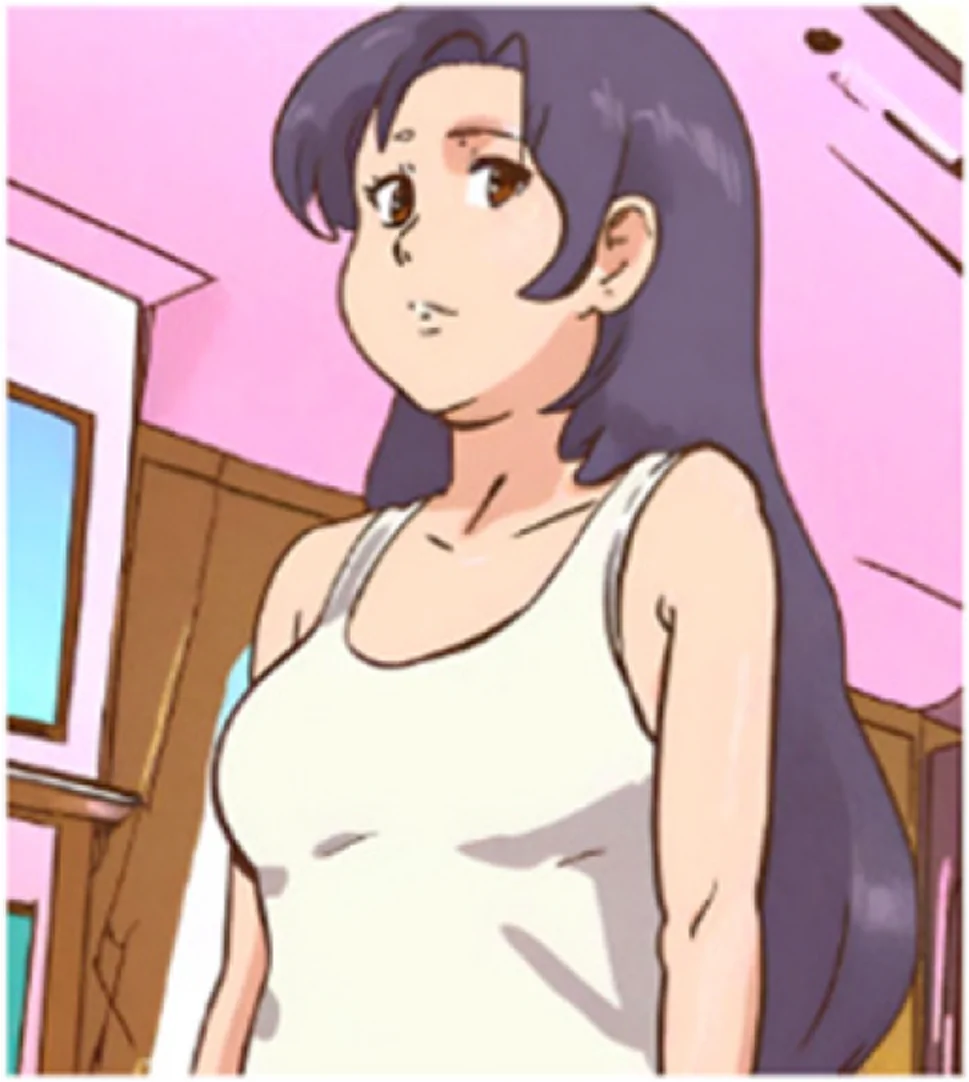
AI-generated image shared by Ashley (20–24 years, Metropolitan, with permission)

*He [MCP] spoke to me with a quiet patronising lilt*,* no explanation just interrogation … When I questioned why he needed to know such things … he retorted that it wasn’t my place to know. I was quite shocked. My care is absolutely my concern. My image conveys my trepidation and lack of expertise/power in the scenario.* (Ashley, 20–24 years, Metropolitan, with Fig. 16)

Women were acutely aware of the unpredictability of their experiences. Where women had positive experiences with one MCP, they also had negative experiences with others.

*Having a different doctor [at] each appointment is quite intimidating as you don’t know how supportive they will be of your birth plan. Things can be smooth sailing and then suddenly you end up with a doctor that starts threatening you with unnecessary intervention. (Tracy*,* 35–39 years*,* Rural)*

## Discussion

The women in our study shared rich and meaningful experiences of maternity care. To our knowledge, this study and our earlier pilot study [18] are the first to use photovoice underpinned by a strengths-based approach to engage larger-bodied women in understanding the impact of and strategies to reduce weight stigma in maternity care. Larger-bodied women experience current approaches in maternity care as weight-centric and stigmatising, characterised by environments and care practices that exclude them, erode their birthing confidence and body image, and create additional financial, time and emotional burdens which can have lasting impacts on their wellbeing. Despite these negative experiences, many women also had contact with individual weight-inclusive MCPs, which shaped their vision for weight-inclusive care.

Numerous previous studies have concluded that weight stigma is common in healthcare [2, 4], including maternity care [3, 8, 9, 11, 12]. Women in this study identified weight-centric care as the mechanism by which stigmatising care was experienced. Maternity care has become increasingly weight-centric, due to the influence of maternity clinical guidelines [34, 35] that are mostly silent on the topic of weight stigma while centring the use of BMI as a marker of health [35, 36]. This is consistent with a recent broader review of obesity-related policies relevant to childbearing women, which found that recognition of and actions to address weight stigma are rare [37].

Current weight-centric maternity clinical guidelines call for larger-bodied women to be routinely counselled before and during their pregnancy about nutrition, exercise, pregnancy risks and the need to limit gestational weight gain [34, 35] Such recommendations reinforce incorrect and stigmatising stereotypes that all larger-bodied women follow a poor diet and are inactive and should therefore be reconsidered. While many women are interested in nutritional advice during pregnancy [38], such advice should be offered to all women (not just those with larger bodies) and linked to wellbeing rather than weight.

While one study, a decade ago, concluded that women wanted more advice about weight during pregnancy [39], our findings raise questions about whether this focus remains appropriate. Studies that have concluded that women want to or at least do not object to being weighed during pregnancy often do not account for the role of internalised weight stigma [19], or the difficulties women may encounter when declining a practice widely regarded by MCPs as benign [40]. For women with a history of body image disturbance or disordered eating, deciding to reject weight as an indicator of their health and wellbeing is common [41, 42]. Many of these women live in larger bodies and the focus on weight in maternity care may be deeply problematic for them. Beyond the negative personal impacts [43], recent evidence suggests that gestational weight gain is not modifiable by behavioural means [44]. This raises questions about the futility of weight monitoring throughout pregnancy, especially given the lack of scholarly attention to weight cycling and weight stigma as contributors to the poorer perinatal outcomes of larger-bodied women [45]. Where discussions about weight and being weighed are clinically relevant, they should proceed only with the woman’s consent.

Participants in this study also perceived that weight-centric care had overshadowed the capacity of individual MCPs and health services to personalise care; a consequence that undermines woman-centredness, a central tenet of contemporary maternity care [46, 47]. Women perceived that repeated counselling about weight and BMI diminished the time available to discuss other topics important to them, and reduced opportunities to facilitate informed decision-making. Other studies have similarly found that larger-bodied women are denied choice and autonomy in maternity care 15, 16, 48], experiencing less partnering communication and shared decision-making opportunities due to weight stigma [14, 48]. Explicit efforts are needed to uphold the rights of larger-bodied women to be involved in decision making about their care.

Women in this study felt they had little autonomy to choose their place of birth, with some women travelling hours to access a hospital that did not exclude them based on BMI. Travelling long distances to access maternity care is associated with poorer perinatal outcomes [49], and providing access to maternity services close to where women live is prioritised by both the World Health Organisation [50] and Australia’s national maternity care strategy [47]. Despite this, larger-bodied women in our study were routinely excluded from maternity services close to their homes, which they perceived to be exclusionary and stigmatising. Place of birth recommendations for all women, including those with larger-bodies, should be made in partnership with the woman, considering an individualised assessment of her health and medical and obstetric history, as well as a careful assessment of the capacity of local health care facilities to provide appropriate care. Greater efforts are also needed to ensure that health services are resourced to meet the needs of their communities.

Many women in this study also reported experiencing only the ‘illusion of choice’ [15] when selecting their model of care. Instead, BMI exclusion criteria, compounded by a lack of shared decision-making, directed women into care pathways that were not their preferred option. Larger-bodied women are often excluded from relationship-based models of care (such as midwifery-led continuity of care, where people receive maternity care from a midwife or small group of midwives throughout pregnant, labour, birth and the postnatal period [51]) despite evidence that these models deliver improved outcomes and experiences for women of all risk, and their babies, as well as providing cost savings to the health system [52, 53]. These models have been identified as the optimal care pathway to support larger-bodied women’s individual pregnancy and birth plans [12]. Expansion of “all risk” midwifery continuity of care models should be prioritised as a key strategy for health services to improve the perinatal outcomes and experiences of larger-bodied women.

Women shared many experiences of feeling excluded or ‘othered’ due to their body size during their maternity care, including a lack of visual representation of larger-bodied women in maternity environments, ill-fitting equipment, and infrastructure. Unaccommodating healthcare environments and a lack of visual representation of body size diversity in media are recognised as facilitators and drivers of weight stigma in maternity care [54]. While other studies have connected weight stigma with disengagement from health services and mistrust of healthcare providers [4, 5], our study extends this to the specific context of maternity care with women sharing experiences of under-reporting symptoms during pregnancy and labour and delayed presentations to maternity care facilities. To realise the potential of midwifery-led continuity of care models for larger-bodied women, health services also need to create weight-inclusive maternity care environments, and provide education that addresses weight-stigmatising attitudes and beliefs of care providers.

Weight-inclusive approaches in healthcare have delivered improvements in physical and mental health outcomes, through models such as Health at Every Size™ [55] and Physical Activity at Every Size [56]. However, none of these models have been applied in the context of maternity care. Women in this study described a clear vision for weight-inclusive maternity care that prioritises individualised, relationship-based care and supports women to work towards a positive and healthy pregnancy, birth and postpartum period. Such an approach is likely to enhance women’s self-efficacy, which participants in the current study perceived to be eroded by weight-centric care. In the context of maternity care, self-efficacy refers to a woman’s belief in her ability to navigate pregnancy, childbirth, and early parenting, including breastfeeding [57]. It is shaped by interactions with care providers, access to supportive resources, and the extent to which care is respectful and empowering. Self-efficacy is known to be an important contributor to wellbeing during pregnancy, birth and postpartum, including breastfeeding [58, 59]. Further research is underway to explore weight-inclusive MCPs’ perspectives and practices, and to co-design nationally agreed best practice principles to address weight stigma in maternity care.

## Strengths and limitations

This study is strengthened by our consumer-led approach, where women with lived experience were authentically involved in all aspects of the research. Our online, pilot-tested approach to photovoice also enabled us to recruit a large participant group, diverse in terms of geographic location, maternity care experiences, age and sexuality. Inclusion of synthesised member checking (involving both participants and lived experience investigators) also strengthened confidence in our findings. Nonetheless, we recognise the views expressed and experiences recounted by the participants may not have been shared by all women. All participants in the study identified as women; the intersection of gender diversity and weight stigma before, during and after pregnancy warrants further study.

## Conclusion

Larger-bodied women experience maternity care as weight-centric and stigmatising, which influences their access to and willingness to seek care. Weight stigma therefore has significant implications for the safety and quality of maternity care for larger bodied women. However, encounters with weight-inclusive MCPs can ameloriate these impacts,, underscoring the need for respectful, individualised maternity care and shared-decision making. Future research should explore the impact of weight stigma on larger-bodied women’s experiences and perinatal outcomes, and build consensus about how to deliver weight-inclusive maternity care.

## Data Availability

The data generated and analysed during the current study are not publicly available due privacy and ethical issues but are available from the corresponding author on reasonable request.
